# Effect of COVID-19 on Musculoskeletal Performance in Gait and the Timed-Up and Go Test

**DOI:** 10.3390/jcm12134184

**Published:** 2023-06-21

**Authors:** Mateusz Kowal, Ewa Morgiel, Sławomir Winiarski, Ewa Gieysztor, Marta Madej, Agata Sebastian, Marcin Madziarski, Nicole Wedel, Krzysztof Proc, Katarzyna Madziarska, Piotr Wiland, Małgorzata Paprocka-Borowicz

**Affiliations:** 1 Department of Physiotherapy, Wroclaw Medical University, T. Chałubińskiego 3, 50-556 Wrocław, Poland; 2 Department of Rheumatology and Internal Medicine, Wroclaw Medical University, Borowska Street 213, 50-556 Wroclaw, Poland; 3 Biomechanics Department, Wroclaw University of Health and Sport Sciences, Paderewskiego 35, 51-612 Wrocław, Poland; 4 Department of Rheumatology and Internal Medicine, University Hospital, Borowska Street 213, 50-556 Wroclaw, Poland; 5 Department of Medicine, Albert Einstein College of Medicine, 1300 Morris Park Ave., New York, NY 10461, USA; 6 Clinical Department of Nephrology and Transplantation Medicine, Wroclaw Medical University, Borowska Street 213, 50-556 Wroclaw, Poland

**Keywords:** symmetry, spatial temporal variables, kinematics, COVID 19, motion analysis, walking, accidentals falls

## Abstract

Introduction: The total number of confirmed cases of COVID-19 caused by the SARS-CoV-2 virus infection is over 621 million in the world. In approximately 63% of cases, the patient still experiences persistent symptoms 30 days after the onset of symptoms or hospitalisation, and 45.9% of patients have experienced or will experience symptoms for at least three months. Despite the prevalence of chronic symptoms and pathological changes that may affect gait and functional mobility in people with a history of COVID-19, there are few publications investigating the impact of these abnormalities. This study aims to determine the long-term effects of COVID-19 on gait and the Timed-Up and Go Task. Material and Methods: A total of 30 individuals took part in the experiment. The subjects in the study group were infected with the COVID-19 virus and required hospital treatment. Prior to the study, the subjects had no chronic diseases or other conditions affecting the musculoskeletal system. The non-infected by COVID-19 group was a healthy population with no history of COVID-19 disease. The study used the inertial system wireless motion analysis system based on 15 inertial sensors (inertial measurement units, IMUs). IMU sensors were placed on the following body segments: head, sternum, middle and lower spine, shoulder, arm, forearm, hand, shank, for the left and right limb. Movement task reports generated from the recording were created using myoRESEARCH 3.10. The subjects in the study group were asked to perform a movement task test—the Timed-Up and Go Test (TUG): sit-to-stand, walk (3 m) without change in direction, walk termination, and stand-to-sit. Results: It took 46% longer for those infected by COVID-19 (participants) to complete the entire movement task compared to those in the not-infected by COVID-19 group. Sit-to-Stand Time [s] was greater in the infected by COVID-19 group and was 2.1 ± 0.7. Mean Walking Speed [m/s] was lower than in the not-infected by COVID-19 group and was 0.26 ± 0.07. Walking cadence [steps/min] was lower and was 21.2 ± 1.2. Infected by COVID-19 participants achieved a smaller anterior pelvic tilt angle (*p* < 0.001) and a smaller hip flexion angle (*p* = 0.025), with an increase in knee (*p* < 0.001) and ankle (*p* < 0.001) flexion angles. Conclusions: Individuals in the infected by COVID-19 group present changes in the ranges of motion and the time to complete the TUG task, despite the fact that at least eight weeks passed after hospital discharge.

## 1. Introduction

To date, a total of more than 621 million confirmed cases of COVID-19 have been identified worldwide [1]. The recovery process for SARS-CoV-2 virus infection causing COVID-19 takes an average of two to three weeks, depending on the severity of symptoms [2,3]. Available studies reveal that 63.2% of patients are still affected 30 days after the onset of symptoms or hospitalisation, and 45.9% still experience the consequences of the disease even after 90 days [4]. A recent definition adopted by World Health Organization (WHO), “post-COVID condition”, refers to the state of persistence of typical symptoms after suspected or confirmed past COVID-19 infection for more than two months, with no other health causes [5]. The post-COVID condition, previously referred to in the literature as “long COVID” or “post-COVID syndrome”, has a wide range of long-lasting physical and psychological symptoms.

These symptoms can be cardiorespiratory (persistent fatigue, dyspnoea), nasopharyngeal (anosmia, sore throat), or neuropsychological (depression, sleep disturbances) [6]. Musculoskeletal symptoms are usually attributed to indirect effects resulting from inflammatory and/or immune responses. Publications also describe post-COVID symptoms that affect the musculoskeletal system (joint and muscle pain) and pathological changes in the constituent tissues and structures as a direct or indirect consequence of SARS-CoV-2 infection [7,8,9,10,11]. COVID-19 can also cause central and peripheral nervous system complications (dizziness, balance problems) [12,13,14]. All these changes reduce functional performance, defined as the ability to perform normal daily living tasks safely and independently, without excessive effort. The basis of functional performance is gait and adopting an upright posture when standing up from a sitting position.

Once acute symptoms have resolved, patients are often referred to rehabilitation units that are dedicated to providing rehabilitation for post-COVID patients. A battery of tests to assess functional performance (sit-to-stand test, Six Minute Walk Test) are used in the examination of the physical performance of individuals with post-COVID disease [15]. Tests prove that normal physical function was detected in only 12% of patients, whereas low, intermediate, and severe impairment was found in 65%, 13%, and 10% of patients, respectively [16,17]. Moreover, changes in gait are also described in case reports, ground reaction forces, treadmill gait studies, and spatial–temporal gait parameters [18,19,20]. Changes in the gait cycle are described using biomechanical parameters.

Human gait consists of load, motion, and cycle [21]. These include changes in speed, gait width, and times of lower limb loadings (spatial and temporal parameters), changes in ranges of motion (kinematic parameters), or ground reaction forces (GRF). Individuals with a history of mild-to-moderate COVID-19 showed more asymmetrical gait patterns than individuals without a history of COVID-19 [19]. Kinetics and muscle activity differ between individuals who recovered from COVID-19 compared with healthy controls [18]. Additionally, post-COVID-19 related symptoms, such as dizziness or depression, affect gait changes and sit-to-stand tasks [22,23].

Despite significant advances in post-COVID research, there are currently not enough studies that analyse the impact of severe COVID-19 on functional performance characteristics. At the same time, most of the available recommendations and rehabilitation programmes seem to focus mainly on improving aerobic capacity and breathing exercises [16,24]. Therefore, this study aims to determine the long-term effects of COVID-19 on gait and the Timed-Up and Go Task. The following research questions need to be answered. Are there differences in the kinematic and temporal variables that characterise the gait of individuals infected by COVID-19 compared to healthy subjects? Are there differences in kinematic and temporal variables that characterise the gait of individuals infected by COVID-19 in the sit-to-stand task? The study findings may provide confirmation of the adequacy of current recommendations, a basis for further analyses, or suggestions for possible changes in the approach to rehabilitation of those infected by COVID-19.

## 2. Material and Methods

### 2.1. Participants

**Figure 1:**
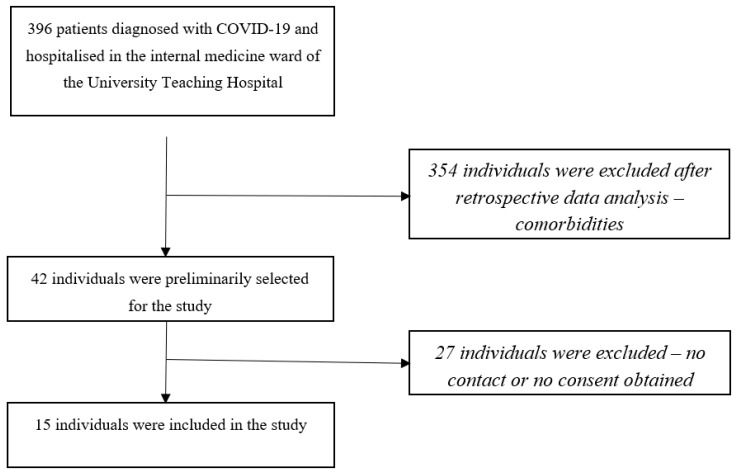
The scheme for recruiting individuals to the study group, consisting of selected patients who were hospitalised in the internal medicine ward of the University Teaching Hospital in Wrocław.

The study group consisted of 15 subjects who were selected from patients hospitalised in the internal medicine department of the University Teaching Hospital from 1 February 2020 to 31 December 2022 with a diagnosis of COVID-19. SARS-CoV2 virus infection was confirmed in all patients by PCR testing. Criteria for hospital admission included age ≥ 18 years and the need for oxygen therapy or pharmacological treatment for in-hospital use (e.g., IV glucocorticosteroids, IV antibiotics, remdesivir, baricitinib, transfusion of blood products). Patients who needed mechanical ventilation and intensive care unit (ICU) care at the time of hospital admission were excluded. Based on a retrospective analysis of hospitalisation data, patients aged 20–50 were systematically selected. They were not burdened with chronic diseases that, in the authors’ opinion, could affect the results of the tests, i.e., history of vascular incidents: stroke, myocardial infarctions, as well as thromboembolism (including the history of venous thrombosis, arterial thrombosis, pulmonary embolism), chronic circulatory failure, chronic respiratory failure, or chronic musculoskeletal diseases. Patients with medical contraindications to exercise load were excluded. Subsequently, phone calls were used to select those willing to participate in the study. After informed consent was obtained, the study was conducted in 15 individuals (Figure 1 recruitment scheme). The non-infected by COVID-19 group consisted of 15 healthy volunteers. Table 1 shows the characteristics of the study group. The Bioethics Committee at Wroclaw Medical University expressed a positive opinion on the study (no. KB-1080/2021).

### 2.2. Experimental Protocol and Setup

The study is best characterized as an observational, cross-sectional study. In the design of our research, we selected two distinct groups: a study group composed of individuals who had infected by the COVID-19 and required hospital treatment, as well as a not-infected by COVID-19 post composed of healthy individuals with no history of COVID-19. A case–control study design was chosen, as it allows for a comparison between the two groups to help determine whether there are differences in the outcomes of the TUG test. It is particularly useful when investigating outcomes (such as motor skills after a COVID-19 infection) that have not been thoroughly researched in the past.

Motion analysis. The Noraxon MyoMotion Research 18 motion analysis system was used for recording raw spatial data. The myoMOTION system is a combination of hardware and software to capture human movement in three degrees of freedom. It consists of a set of 16 sensors using inertial sensor technology. Based on the so-called fusion algorithms (sensor fusion algorithm), data from the three-dimensional accelerometer, gyroscope, and magnetometer are used for measuring the three-dimensional rotation angles of each sensor in absolute space (vertical tilt, also known as orientation or navigation angles). The Myomotion inertial motion unit system from Noraxon appears to be a highly valid, accurate, repeatable, and reliable tool for measuring human motion. Proper consideration of error and tolerance is important in order to ensure the most accurate and informative results [25].

**Figure 2:**
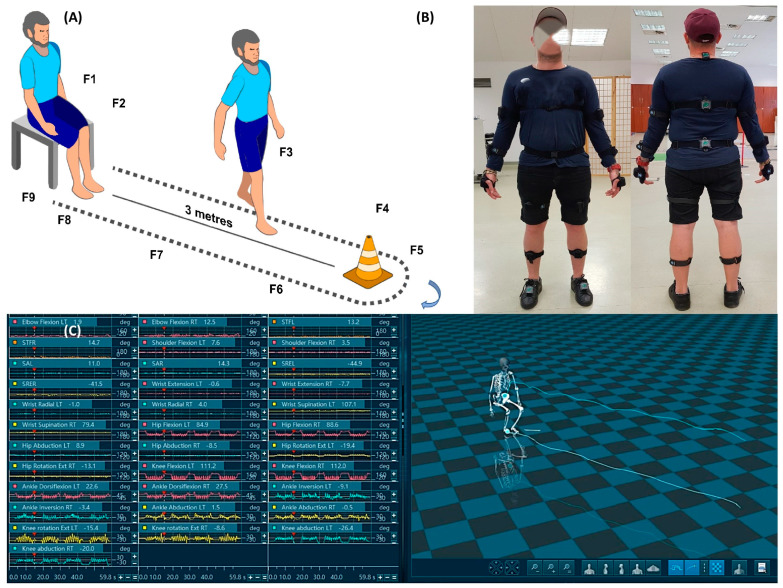
(**A**) Movement sequences of the analysed TUG test: in order from left: sit-to-stand (F1), walking initiation (F2), walk (F3), walk termination (F4), turn (F5), walking initiation (F6), walk (F7), walk termination (F8), and turn and taking a sitting position in a chair (stand-to-sit) (F9); (**B**) diagram of the placement of the IMU sensors on the participant’s body: (**C**) an example of the raw data recorded during the test.

The position of the sensors is shown in Table 2. It corresponds to the body segments (Figure 2B).

Each sensor was carefully positioned in order to provide optimal data collection for each body segment’s motion in three degrees of freedom. These placements ensure that the full range of human body motion can be captured accurately, with each sensor providing data on specific body segment movements and rotations in three dimensions. The placement of the sensors was conducted as per the recommended guidelines of Noraxon, ensuring a standardized and reliable procedure.

Division into phases (sub-tasks) and motion cycles. The TUG test is one of the basic clinimetric tests and consists of several critical and consecutive movement sequences (Figure 1). The first phase (F1) is standing up from a chair (sit-to-stand), the second phase (F2) is walking initiation, then (F3) walking at a relatively constant speed (walk), (F4) stopping, i.e., walk termination, (F5) turning (turn), (F6) another walking initiation, (F7) walk, (F8) walk termination, and, finally, (F9) a turn and taking a sitting position in a chair (stand-to-sit) [26]. For the purpose of this article, the temporal characteristics of the entire movement task of the TUG test and the two most repetitive phases, i.e., Sit-to-Stand and Walk during forward movement and a turn, were selected to classify the movement dynamics. Each movement task was repeated four times, and the phases and motion cycles were averaged.

### 2.3. Data Analysis

The participant was asked to stand up from a chair without the help of the upper limbs, walk to a cone, go around it, return, and sit down in a chair. The person started the movement with any lower limb and went around the cone 3 times from the right side and 3 times from the left side. Phases were divided based on the steps performed (using the “Contact” function in the MR3 software). The TUG test begins with the Sit-to-Stand phase (F1), a transition from seated to standing that necessitates overcoming gravity and establishing balance. This segues into the Walking Initiation phase (F2), where individuals shift their body weight and take initial steps. The Walk phase (F3) follows, characterized by a relatively constant speed and steady-state balance. This leads to the Walk Termination phase (F4), where individuals decelerate, and balance control is emphasized.

A change in direction occurs in the Turn phase (F5), demanding dynamic balance and coordination. Another Walking Initiation phase (F6) ensues, similar to F2, but post-turn, presenting unique balance challenges. The process again enters a Walk phase (F7), identical to F3, and a Walk Termination phase (F8), mirroring F4.

Finally, the sequence concludes with the Stand-to-Sit phase (F9), where individuals transition from standing to seated, requiring a controlled lowering of the body against gravity. The prerformed study focused on the entire movement task and the two most repetitive phases, i.e., Sit-to-Stand and Walk during forward movement and a turn, as they represent key aspects of mobility and balance and are commonly affected in individuals with mobility impairment. The Timed-Up and Go test was validated for the Polish population [27]. Angle-time series were averaged over 4 trials and reported against normalised time. The movement analysis was limited to the assessment of gait time series defining movement of the pelvis and movement of the lower limb joints in the sagittal plane. The Sit-to-Stand task was divided into four corresponding movement phases in the transition from a sitting to a standing position according to Schenkmann [28]. The definition of anatomical angles follows the rules and regulations of the neutral medical/null method. In accordance with the ISB recommendations for the definition of a joint coordinate system, the following angles were selected for the reporting of human joint movement, sampled every 0.1% of the duration of the sit-to-stand phase or walk phase and normalised to the phase duration (% time) [29,30]:

Pelvic tilt: anterior (positive) or posterior (negative) movement of the pelvis in the sagittal plane;
Hip flexion/extension: positive (flexion) or negative (extension) movement of the femur in the sagittal plane;
Knee flexion/extension: positive (flexion) or negative (extension) movement of the femur in the sagittal plane;
Ankle dorsiflexion/plantarflexion: positive (dorsi) or negative (plantar) movement of the foot in relation to the tibia in the sagittal plane.

### 2.4. Statistical Analysis

In this retrospective comparative study, descriptive statistics were performed by calculating median values and the interquartile range (IQR) value as measures of deviation from the mean. The IQR is a measure of variability and statistical dispersion, being equal to the difference between the 75th and 25th percentiles, or between upper and lower quartiles. A framework for the definition of standardised protocols for measuring kinematics was established, and a sample size was estimated using the recommendations postulated by Kontaxis et al. [31]. The statistical power was sufficient to detect the differences described.

The normal distribution of the variables was verified using the W Shapiro-Wilk test. In cases where the W statistic was significant, the hypothesis that the sample analysed was from a normal distribution was rejected. Differences in parameters between the results of experimental and not-infected by COVID-19 measurements were checked using the Student’s *t*-test in groups of equal size for intergroup comparisons or the Mann-Whitney U test in the event of non-compliance with normal distribution. All statistical calculations were performed using Statistica 13.1 (TIBCO Software Inc., Stanford, CA, USA).

Differences between continuous variables (time series) were analysed using the One-Dimensional Statistical Parametric Mapping (SPM1D) test. Statistical Parametric Mapping (SPM) is a technique for the statistical analysis of typical biomechanical data, e.g., 1D curves and vectors. The SPM works similarly to basic statistical analyses; however, it extends analyses to one-dimensional force profiles or kinematics. SPM1D in the Python programming language is a one-dimensional statistical parametric mapping package and uses Random Field Theory’s expectations of smooth, one-dimensional (random) Gaussian fields for statistical inference over a set of one-dimensional measurements. The SPM procedure also takes into account the alignment, normalisation, and smoothing of the time series data. Parametric statistical models are established in each voxel using a General Linear Model to describe the data in terms of experimental and disturbance effects and residual variability.

For each participant and time-dependent angular data, a 2-sample *t*-test of SPM [6] was numerically calculated (with alpha = 0.05, non-sphericity correction and assumption of unequal variances) [32]. A statistical map was created for each test by calculating the conventional univariate t-statistic at each point of the curve. If the SPM exceeded a preset threshold, an additional threshold cluster was created to indicate a significant difference (grey area) between the two compared joint movement patterns at a specific point in the gait cycle [33].

## 3. Results

For the extracted data and for significant changes (alpha = 0.05), the partial effect size η^2^ was found to range from 0.67 to 0.88. All variables are significantly different between the groups (Table 2). The gait speed is relatively low in the experimental and not-infected by COVID-19. The Sit-to-Stand task alone took 36% longer to complete in the infected by COVID-19 group compared to the healthy subjects. In the speed parameters tested, those in the infected by COVID-19 group achieved a 46% lower gait speed and a 42% lower speed for the entire TUG task (Table 3).

**Figure 3:**
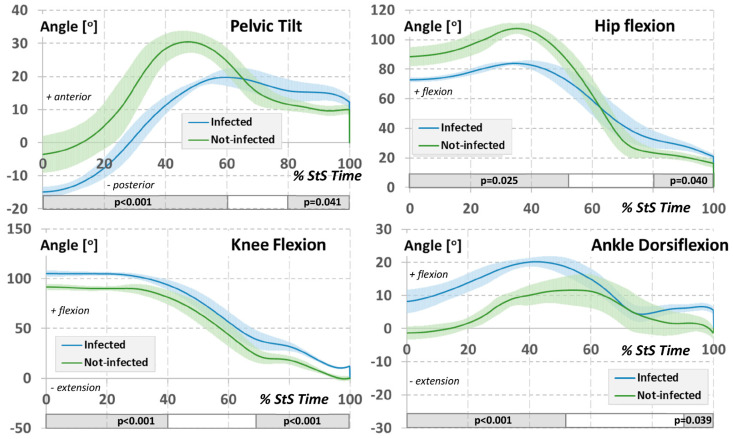
Averaged waveforms of the lower limb and pelvic joint angular variables in the sagittal plane for the sit-to-stand movement task for experimental groups. The colour-shaded area designates ±1 standard deviation from the mean. The rectangular, shaded area under each graph includes the significant results of the SPM statistical difference test.

The analysis of the lower limb and pelvic joint angular variables in the sagittal plane recorded several differences in terms of the Sit-to-Stand task between the groups (Figure 3). Subjects in the study group from the initiation of the standing-up movement until the transition to the upright position achieved a smaller anterior pelvic tilt angle (*p* < 0.001) and a smaller hip flexion angle (*p* = 0.025) with an increase in knee (*p* < 0.001) and ankle (*p* < 0.001) flexion angles.

In the analysis of lower limb and pelvic joint angular variables in the sagittal plane during the Sit-to-Stand task, our results depicted several notable differences between the two groups (Figure 3). Specifically, the study group, from the commencement of the standing-up movement to the transition to the upright position, demonstrated a smaller anterior pelvic tilt angle (*p* < 0.001) and a lesser hip flexion angle (*p* = 0.025). This suggests that the study group had a more challenging time achieving the full upright position as compared to the non-infected by COVID-19 group. Moreover, the study group displayed increased knee (*p* < 0.001) and ankle (*p* < 0.001) flexion angles, implying a potential compensatory strategy to manage the task.

**Figure 4:**
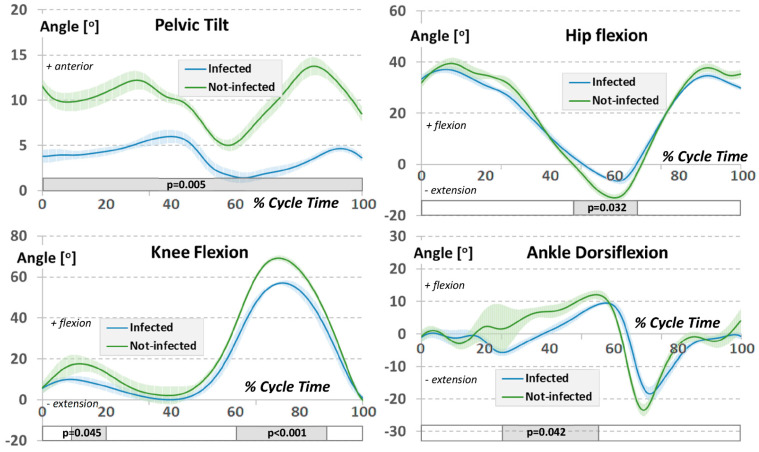
Averaged waveforms of the lower limb and pelvic joint angular variables in the sagittal plane for the gait cycle for both experimental groups. The colour-shaded area designates ±1 standard deviation. The rectangular, shaded area under each graph includes the significant results of the SPM statistical difference test.

Furthermore, the lower limb and pelvic joint angular variables in the sagittal plane revealed substantial differences in gait between the two groups (Figure 4). The study group maintained a significantly higher posterior pelvic tilt throughout the gait cycle compared to the non-infected by COVID-19 group (*p* = 0.005). This might suggest a less efficient or more strenuous gait pattern in the study group. The hip joint range of motion was predominantly symmetrical and akin to the not-infected by COVID-19, barring the final phase of support, where the study group’s hip joint was less flexed (*p* = 0.032), indicating potential difficulties in completing the gait cycle.

Similarly, the range of flexion movement at the knee joint during the swing phase was significantly lower in the study group compared to the non-infected by COVID-19 group (*p* < 0.001), indicating that the study group may have difficulties in leg advancement during the swing phase. Lastly, the study group’s ankle joint range of motion during gait showed significant differences in the middle phase of support, where it was less flexed compared to the non-infected by COVID-19 group (*p* = 0.042). This suggests that the study group may have issues with foot clearance and heel strike, both integral parts of a smooth and efficient gait cycle. Taken together, our results indicate that the study group displayed a significantly worse performance in walking when compared to the non-infected by COVID-19 group.

## 4. Discussion

Undertaking this study was mainly driven by the need to take a closer look at those infected by COVID-19, specifically their musculoskeletal symptoms, and it was based on the obtained results, which suggest possible changes in the approach to rehabilitation of those infected by COVID-19. More specifically, this study aimed to determine the long-term effects of COVID-19 on gait and the TUG task. This study found that individuals in the infected by COVID-19 group presented changes in the ranges of motion and time to complete the TUG task, despite at least eight weeks passing after their hospital discharge, suggesting that COVID-19 infection can impact musculoskeletal performance.

The use of inertial measures as an instrument to assess those infected by COVID-19, specifically their musculoskeletal symptoms, is a novel approach. This study used a system based on inertial sensors (IMUs), which are increasingly used for investigating patients’ clinical status and treatment effects. IMU sensors’ use in analysing the movement of people with internal diseases is rare and relatively new [34]. It should be noted that the IMU sensor-based system has also successfully passed several validation checks in which the analysis results were compared to the gold standard, the optoelectronic motion analysis system [25,35,36]. Currently, there is only a limited number of studies on the directions of compensation in individuals infected by COVID-19. The study also pointed out the limited number of studies on compensation directions in individuals with post-COVID conditions, despite the prevalence of the condition.

The first stage in the data analysis was an attempt to find differences and temporal variables that characterise the gait and sit-to-stand of individuals infected by COVID-19 compared with healthy subjects. Compared to the free gait of healthy subjects in the general population, both infected by COVID-19 and not-infected by COVID-19 walked more slowly; however, in the TUG test, the gait is over a relatively short distance, where speeds are not as high as on laboratory paths [37,38]. It took 46% longer for those infected by COVID-19 (participants) to complete the entire movement task compared to those in the non-infected by COVID-19 group. The results obtained by individuals in the infected by COVID-19 group indicate a significant impact of past infection on the increased risk of falling. A score of more than 10 s is considered to indicate an increased risk of falling [39]. The participants in the infected by COVID-19 group scored 16.5 ± 0.9 s. The individuals in the infected by COVID-19 group were subjected to rigorous inclusion options (e.g., lack of comorbidities), and despite the passage of time, the changes are still noticeable. In available studies, Keklicek et al. showed significantly more significant lower limb asymmetry in the duration of the double-support phase (*p* = 0.042), single-support phase (*p* = 0.006), and weight-bearing phase (*p* = 0.042) in the mild/moderate COVID-19 group [19]. Keklicek et al. consider a possible effect of infection on the central and peripheral nervous system as a potential cause of the variation in gait symmetry [19,38]. Another difference that may affect the results of the TUG task is the higher BMI in the infected by COVID-19 group. Among 148,494 U.S. adults with COVID-19, a nonlinear relationship was found between body mass index (BMI) and COVID-19 severity, with lowest risks at BMIs near the threshold between healthy weight and overweight in most instances, which then increased with higher BMI [40]. Being overweight and obesity were risk factors for invasive mechanical ventilation [41]. In the infected by COVID-19 group, mechanical ventilation was not included in the therapy; however, the severity of the disease could be an additional factor to consider, which may affect the TUG test results.

### 4.1. Repercussions of COVID-19 on Aerobic Performance and Lung Function

In individuals with COVID-19, particularly those who required oxygen assistance, there is a complex interplay between cardiorespiratory function and gait performance. Cardiorespiratory function has been shown to correlate with the severity of persistent symptoms in post-COVID-19 condition, where greater physical fitness and cardiopulmonary function are associated with a lower severity of symptoms. Notably, cardiorespiratory fitness, lower-limb muscle strength, maximal voluntary ventilation, and left ventricular ejection fraction can contribute to reducing fatigue and dyspnea, common symptoms among these patients. As such, the value of exercise and physical conditioning as pre- and post-COVID-19 countermeasures is evident, as they could help decrease the severity of both acute infection and persistent post-COVID-19 symptoms [42]. Importantly, even mild to moderate COVID-19 can impact cardiorespiratory fitness in the mid-term, as shown in a study on elite athletes. These individuals reached the aerobic threshold earlier than controls, indicating a possible need for a longer period of readjustment to aerobic exercise post-COVID-19. It is worth noting, however, that other cardiorespiratory fitness parameters did not differ significantly between the groups, nor were there detectable differences in resting pulmonary and cardiac examinations [43].

### 4.2. Repercussions of COVID-19 on Muscle Strength and Fatigue

Musculoskeletal investigation plays a crucial role in understanding human movement and biomechanics, which are essential for the development of medical implants, such as hip replacements [44,45]. The second stage of the analysis was to search for differences in kinematics, which characterise the gait and Sit-to-Stand of people infected by COVID-19 in the Sit-to-Stand task. Compared to those not infected by COVID-19, the individuals in the infected by COVID-19 group exhibited asymmetric positioning of the pelvis, expressed as a reduced anterior tilt angle. This change was statistically significant in both the Sit-to-Stand and gait tasks. It should be noted that the range of pelvic movement in the sagittal plane is not great, ranging from 6° to 10° under physiological conditions. In the infected by COVID-19 group, changes in pelvis positioning were accompanied by changes in the range of motion of the thigh at the hip joint. Again, the range of motion changes were related to both movement tasks tested. During Sit-to-Stand and gait tests, the thigh at the hip joint reached a smaller angle of extension. The most significant asymmetry concerned the transition from the sitting position to the upright position in the Sit-to-Stand task and the end of the support phase in gait. In both of these cases, the involvement of the hip extensors is greatest. So far, the authors have not found a similar study with which the results could be compared. However, some publications report the effects of SARS-CoV-2 virus infection on the muscular system. In a study by Halpin et al. of patients treated for a severe course of COVID-19, 60.3% of patients complained of increased fatigue, despite not having had COVID-19 infection for 48 days [16]. These individuals who were treated required additional care during their hospital stay compared to those who were not infected [46].

A study of factors affecting physical performance after cessation of acute COVID-19 revealed that the age of patients, comorbidities, and the need for mechanical ventilation influenced the increased need for rehabilitation after discharge [15]. A study by Yamada et al. reports that patients with increased risk factors had a 63% rate of being able to walk unaided at discharge [38]. In our study, the individuals in the study group were young and had no comorbidities, yet compensatory movements were recorded. Asymmetry in the way lower limbs were loaded after COVID-19 infection was also recorded by Jafarnezhadgero et al. in subjects who were recreational runners [18]. In a case report of a 48-year-old COVID-19 patient with neurological impairment and gait difficulties, Pistoia et al. attributed the cause to a systemic inflammatory process [6]. In terms of gait performance, studies have shown that individuals with a history of COVID-19 may exhibit asymmetric gait patterns, even after full recovery. The severity of the asymmetric gait pattern may be influenced by post-COVID symptoms which include dizziness, balance disorders, and changes in the peripheral nervous system. This highlights the potential long-term neuro–motor impact of the disease, regardless of its initial severity, and it emphasises the importance of detailed clinical follow-up to monitor and manage these effects.

Another important factor affecting the results obtained in our study group may have been muscle weakness. Patients after COVID-19 suffer from skeletal muscle weakness and exercise intolerance. Histological sections present muscle fibre atrophy, metabolic alterations, and immune cell infiltration [47]. Paneroni et al. assessed the effect of COVID-19 infection on muscle strength in subjects who, similar to those in our study group, were healthy before the disease. They found that the quadriceps were weak in 86% of subjects, and biceps were weak in 73% of subjects [48]. A decrease in muscle strength is often observed in hospitalized patients and is associated with bed rest. However, the publication by Floreani et al., which investigated the effects of 14 days of bed rest and following physical training on metabolic cost, mechanical work, and efficiency during walking in older and young healthy males, showed that 14 days of bed rest does not affect gait [49]. Our study included subjects aged 37.6 ± 6.1 who were enrolled a minimum of eight weeks prior to measurement. For this reason, we assumed that the effect of bed rest was reduced.

### 4.3. Study Limitation

This study has some limitations that need to be considered when interpreting the results. Firstly, this study was conducted in a single clinical center, which may limit the generalizability of the findings to other settings. Secondly, the small sample size does not allow extending the follow-up to all patients after COVID-19 to draw definitive conclusions. The provisional endorsement of the TUG test as a screening assessment in the long term must be considered on a case-by-case evaluation by the treatment team. Additional limitations of the study were the differences in BMI and age of the subjects in the study group, which may affect the results of this study [50]. As such, caution should be exercised when interpreting the results and further research with larger sample sizes and more diverse populations is needed to confirm and extend these findings.

### 4.4. Conclusions

Individuals in the infected by COVID-19 group presented changes in the ranges of motion and the time to complete the TUG task, even though at least eight weeks passed after hospital discharge. Further research into the strength capabilities of large muscle groups of people with post-COVID would help determine post-infectious fatigue rates’ roles. The results of the TUG analysis in those infected by COVID-19 should be considered on a more individual basis. The recorded changes in the post- group with infected by COVID-19 symptoms and without a significant past medical history indicate that movement patterns deviate from normative values despite the passage of time.

## Figures and Tables

**Table 1:** Characteristics of the study participants (mean ± SD).

Characteristics	Infected by COVID-19 (*n* = 15)	Not-Infected by COVID-19 (*n* = 15)
Age [years]	37.6 ± 6.1	32.3 ± 4.0
Male/female	12/3	10/5
Height [cm]	177.2 ± 0.1	177.4 ± 10.0
BMI [kg/m^2^]	28.5 ± 4.2	25.57 ± 2.1
COVID-19-associated pneumonia in imaging tests	15	N/A
Oxygen therapy during hospitalization	11	N/A
Comorbidities	2 (hypothyroidism-1, insulin resistance-1)	N/A
Number of days of hospitalisation	(4–12)	N/A
Time interval between end of hospitalisation and conduct of the study in weeks	(8–25)	N/A
Symptoms reported by patients in relation to COVID-19	-	N/A
- fatigue	5	
- muscle pain	3	
- joint pain	5	

Abbreviations: Infected by COVID-19, SD: standard deviation; n: number of participants, BMI: body mass index; N/A: not applicable.

**Table 2:** The position of the sensors on the body of the tested individual.

Region	Inertial Meter Positions
Head	The sensor is attached to a headband on the center of the forehead.
Sternum	The sensor is placed on the chest, usually at the level of the sternum or breastbone.
Mid-spine	This sensor is placed around the mid-point of the spine, typically around the level of the thoracic vertebrae.
Lower spine	A sensor is affixed at the lumbar region of the spine.
Left/Right shoulder	One sensor is placed on each shoulder, typically on the acromion—the bony prominence at the top of the shoulder.
Left/Right upper arm	Sensors are attached on the lateral side of each upper arm, usually midway between the shoulder and the elbow.
Left/Right forearm	Sensors are positioned on the lateral side of each forearm, typically halfway between the elbow and the wrist.
Left/Right hand	A sensor is placed on the dorsum (back) of each hand, usually near the wrist.
Left/Right thigh	Sensors are attached to each thigh, typically midway between the hip and knee, on the lateral side.
Left/Right shank	Sensors are placed on the lateral side of each shank, usually midway between the knee and the ankle.

**Table 3:** Results for time variables that characterise the TUG task and its phases. The experimental group of individuals from the infected by COVID-19 group ±SD.

	Infected by COVID-19	Not-Infected by COVID-19	*p*-Value (*t*-Test/U-Test)
TUG Time [s]	16.5 ± 0.9	11.3 ± 0.7	<0.01
Sit-to-Stand Time [s]	2.1 ± 0.7	1.6 ± 0.5	<0.01 ^U^
Walking Cycle Time [s]	5.7 ± 0.8	4.5 ± 0.7	<0.01 ^U^
Mean Speed [m/s]	0.18 ± 0.05	0.31 ± 0.01	<0.01 ^U^
Mean Walking Speed [m/s]	0.26 ± 0.07	0.48 ± 0.09	<0.01 ^U^
Walking Cadence [steps/min]	21.2 ± 1.2	32.8 ± 1.5	<0.01

^U^—designation of the Mann-Whitney U test in the event of non-compliance with normal distribution.

## Data Availability

The data presented in this study are available on request from the corresponding author.

## References

[1] World Health Organization Coronavirus Disease (COVID-19) Pandemic. www.who.int/emergencies/diseases/novel-coronavirus-2019.

[2] Daher A., Balfanz P., Cornelissen C., Müller A., Bergs I., Marx N., Müller-Wieland D., Hartmann B., Dreher M., Müller T. (2020). Follow up of patients with severe coronavirus disease 2019 (COVID-19): Pulmonary and extrapulmonary disease sequelae. Respir. Med..

[3] Sudre C.H., Murray B., Varsavsky T., Graham M.S., Penfold R.S., Bowyer R.C., Pujol J.C., Klaser K., Antonelli M., Canas L.S. (2021). Attributes and predictors of long COVID. Nat. Med..

[4] Fernández-De-Las-Peñas C., Palacios-Ceña D., Gómez-Mayordomo V., Florencio L.L., Cuadrado M.L., Plaza-Manzano G., Navarro-Santana M. (2021). Prevalence of post-COVID-19 symptoms in hospitalized and non-hospitalized COVID-19 survivors: A systematic review and meta-analysis. Eur. J. Intern. Med..

[5] Soriano J.B., Murthy S., Marshall J.C., Relan P., Diaz J.V. (2022). Definition WHOCC. A clinical case definition of post-COVID-19 condition by a Delphi consensus. Lancet Infect. Dis..

[6] Aiyegbusi O.L., Hughes S.E., Turner G., Rivera S.C., McMullan C., Chandan J.S., Haroon S., Price G., Davies E.H., Nirantharakumar K. (2021). Symptoms, complications and management of long COVID: A review. J. R. Soc. Med..

[7] Barker-Davies R.M., O’Sullivan O., Senaratne K.P.P., Baker P., Cranley M., Dharm-Datta S., Ellis H., Goodall D., Gough M., Lewis S. (2020). The Stanford Hall consensus statement for post-COVID-19 rehabilitation. Br. J. Sport. Med..

[8] Ramani S.L., Samet J., Franz C.K., Hsieh C., Nguyen C.V., Horbinski C., Deshmukh S. (2021). Musculoskeletal involvement of COVID-19: Review of imaging. Skelet. Radiol..

[9] Kucudiaeresi A. (2022). Management of musculoskeletal consequences in persons with post-COVID-19 syndrome. Aging Clin. Exp. Res..

[10] Disser N.P., De Micheli A.J., Schonk M.M., Konnaris M.A., Piacentini A.N., Edon D.L., Toresdahl B.G., Rodeo S.A., Casey E.K., Mendias C.L. (2020). Musculoskeletal Consequences of COVID-19. J. Bone Jt. Surg. -Am. Vol..

[11] Rosa K.Y.A., Padua K.L.C., Maldaner V.Z., de Oliveira L.V.F., de Melo F.X., Santos D.B. (2021). Musculoskeletal Consequences from COVID-19 and the Importance of Pulmonary Rehabilitation Program. Respiration.

[12] Song W.-J., Hui C.K.M., Hull J.H., Birring S.S., McGarvey L., Mazzone S.B., Chung K.F. (2021). Confronting COVID-19-associated cough and the post-COVID syndrome: Role of viral neurotropism, neuroinflammation, and neuroimmune responses. Lancet Respir. Med..

[13] (2021). Long COVID: Understanding the neurological effects. Lancet Neurol..

[14] Heneka M.T., Golenbock D., Latz E., Morgan D., Brown R. (2020). Immediate and long-term consequences of COVID-19 infections for the development of neurological disease. Alzheimers Res. Ther..

[15] Paneroni M., Vogiatzis I., Bertacchini L., Simonelli C., Vitacca M. (2021). Predictors of Low Physical Function in Patients With COVID-19 With Acute Respiratory Failure Admitted to a Subacute Unit. Arch. Phys. Med. Rehabil..

[16] Halpin S.J., McIvor C., Whyatt G., Adams A., Harvey O., McLean L., Walshaw C., Kemp S., Corrado J., Singh R. (2021). Postdischarge symptoms and rehabilitation needs in survivors of COVID-19 infection: A cross-sectional evaluation. J. Med. Virol..

[17] Carfi A., Bernabei R., Landi F., Gemelli Against C.-P.A. (2020). Persistent Symptoms in Patients After Acute COVID-19. Jama J. Am. Med. Assoc..

[18] Jafarnezhadgero A.A., Hamlabadi M.P., Sajedi H., Granacher U. (2022). Recreational runners who recovered from COVID-19 show different running kinetics and muscle activities compared with healthy controls. Gait Posture.

[19] Keklicek H., Selcuk H., Kurt I., Ulukaya S., Ozturk G. (2022). Individuals with a COVID-19 history exhibit asymmetric gait patterns despite full recovery. J. Biomech..

[20] Pistoia F., Ornello R., Sucapane P., Marini C., Sacco S. (2021). Symptoms of gait and coordination impairment in a patient with COVID-19 interstitial pneumonia. Neurol. Sci..

[21] Jamari J., Ammarullah M.I., Santoso G., Sugiharto S., Supriyono T., Permana M.S., Winarni T.I., van der Heide E. (2022). Adopted walking condition for computational simulation approach on bearing of hip joint prosthesis: Review over the past 30 years. Heliyon.

[22] Fraser J.J., VanDehy J., Bodell D.M., Gottshall K.R., Sessoms P.H. (2021). Head and Body Dyskinesia During Gait in Tactical Athletes With Vestibular Deficit Following Concussion. Front. Sport. Act. Living.

[23] Kumar D., Villarreal D.J., Meuret A.E. (2021). Walking on the bright side: Associations between affect, depression, and gait. PLoS ONE.

[24] Thomas P., Baldwin C., Bissett B., Boden I., Gosselink R., Granger C.L., Hodgson C., Jones A.Y., E Kho M., Moses R. (2020). Physiotherapy management for COVID-19 in the acute hospital setting: Clinical practice recommendations. J. Physiother..

[25] Park S., Yoon S. (2021). Validity Evaluation of an Inertial Measurement Unit (IMU) in Gait Analysis Using Statistical Parametric Mapping (SPM). Sensors.

[26] Li T., Chen J., Hu C., Ma Y., Wu Z., Wan W., Huang Y., Jia F., Gong C., Wan S. (2018). Automatic Timed Up-and-Go Sub-Task Segmentation for Parkinson’s Disease Patients Using Video-Based Activity Classification. IEEE Trans. Neural Syst. Rehabil. Eng..

[27] Wisniowska-Szurlej A., Cwirlej-Sozanska A., Woloszyn N., Sozanski B., Wilmowska-Pietruszynska A., Washburn R. (2020). Cultural adaptation and validation of the Polish version of the physical activity scale for older people living in a community: A cross-sectional study. Eur. Rev. Aging Phys. Act..

[28] Schenkman M., Berger R.A., Riley P.O., Mann R.W., Hodge W.A. (1990). Whole-Body Movements during Rising to Standing from Sitting. Phys. Ther..

[29] Wu G., Siegler S., Allard P., Kirtley C., Leardini A., Rosenbaum D., Whittle M., D’Lima D.D., Cristofolini L., Witte H. (2002). ISB recommendation on definitions of joint coordinate system of various joints for the reporting of human joint motion-part 1: Ankle, hip, and spine. J. Biomech..

[30] Wu G., van der Helm F.C.T., Veeger H.E.J., Makhsous M., Van Roy P., Anglin C., Nagels J., Karduna A.R., McQuade K., Wang X. (2005). ISB recommendation on definitions of joint coordinate systems of various joints for the reporting of human joint motion-Part II: Shoulder, elbow, wrist and hand. J. Biomech..

[31] Kontaxis A., Cutti A.G., Johnson G.R., Veeger H.E.J. (2009). A framework for the definition of standardized protocols for measuring upper-extremity kinematics. Clin. Biomech..

[32] Bankosz Z., Winiarski S. (2021). The Application of Statistical Parametric Mapping to Evaluate Differences in Topspin Backhand between Chinese and Polish Female Table Tennis Players. Appl. Bionics Biomech..

[33] Bankosz Z., Winiarski S. (2020). Statistical Parametric Mapping Reveals Subtle Gender Differences in Angular Movements in Table Tennis Topspin Backhand. Int. J. Environ. Res. Public Health.

[34] Saito Y., Ishida T., Kataoka Y., Takeda R., Tadano S., Suzuki T., Nakamura K., Nakata A., Osuka S., Yamada S. (2022). Evaluation of gait characteristics in subjects with locomotive syndrome using wearable gait sensors. BMC Musculoskelet. Disord..

[35] Mundt M., Thomsen W., David S., Dupré T., Bamer F., Potthast W., Markert B. (2019). Assessment of the measurement accuracy of inertial sensors during different tasks of daily living. J. Biomech..

[36] Bankosz Z., Winiarski S., Lanzoni I.M. (2020). Gender Differences in Kinematic Parameters of Topspin Forehand and Backhand in Table Tennis. Int. J. Environ. Res. Public Health.

[37] Pietraszewski B., Winiarski S., Jaroszczuk S. (2012). Three-dimensional human gait pattern-reference data for normal men. Acta Bioeng. Biomech./Wroc. Univ. Technol..

[38] Winiarski S., Pietraszewska J., Pietraszewski B. (2019). Three-Dimensional Human Gait Pattern: Reference Data for Young, Active Women Walking with Low, Preferred, and High Speeds. Biomed Res. Int..

[39] Shumway-Cook A., Brauer S., Woollacott M. (2000). Predicting the probability for falls in community-dwelling older adults using the Timed Up & Go Test. Phys. Ther..

[40] Kompaniyets L., Goodman A.B., Belay B., Freedman D.S., Sucosky M.S., Lange S.J., Gundlapalli A.V., Boehmer T.K., Blanck H.M. (2021). Body Mass Index and Risk for COVID-19-Related Hospitalization, Intensive Care Unit Admission, Invasive Mechanical Ventilation, and Death-United States, March-December 2020. Mmwr-Morb. Mortal. Wkly. Rep..

[41] Kollias A., Kyriakoulis K.G., Syrigos K. (2021). Obesity and Mortality Among Patients Diagnosed With COVID-19. Ann. Intern. Med..

[42] Jimeno-Almazán A., Martínez-Cava A., Buendía-Romero Á., Franco-López F., Sánchez-Agar J.A., Sánchez-Alcaraz B.J., Tufano J.J., Pallarés J.G., Courel-Ibáñez J. (2022). Relationship between the severity of persistent symptoms, physical fitness, and cardiopulmonary function in post-COVID-19 condition. A population-based analysis. Intern. Emerg. Med..

[43] Anastasio F., LA Macchia T., Rossi G., D’abbondanza M., Curcio R., Vaudo G., Pucci G. (2022). Mid-term impact of mild-moderate COVID-19 on cardiorespiratory fitness in elite athletes. J. Sport. Med. Phys. Fit..

[44] Jamari J., Ammarullah M.I., Saad A.P.M., Syahrom A., Uddin M., van der Heide E., Basri H. (2021). The Effect of Bottom Profile Dimples on the Femoral Head on Wear in Metal-on-Metal Total Hip Arthroplasty. J. Funct. Biomater..

[45] Ammarullah M.I., Hartono R., Supriyono T., Santoso G., Sugiharto S., Permana M.S. (2023). Polycrystalline Diamond as a Potential Material for the Hard-on-Hard Bearing of Total Hip Prosthesis: Von Mises Stress Analysis. Biomedicines.

[46] Grove K., Edgar D.W., Chih H., Harrold M., Natarajan V., Mohd S., Hurn E., Cavalheri V. (2022). Greater In-Hospital Care and Early Rehabilitation Needs in People with COVID-19 Compared with Those without COVID-19. J. Clin. Med..

[47] Soares M.N., Eggelbusch M., Naddaf E., Gerrits K.H.L., van der Schaaf M., van den Borst B., Wiersinga J., van Vugt M., Weijs P.J.M., Murray A.J. (2022). Skeletal muscle alterations in patients with acute COVID-19 and post-acute sequelae of COVID-19. J. Cachexia Sarcopenia Muscle.

[48] Paneroni M., Simonelli C., Saleri M., Bertacchini L., Venturelli M., Troosters T., Ambrosino N., Vitacca M. (2021). Muscle Strength and Physical Performance in Patients Without Previous Disabilities Recovering From COVID-19 Pneumonia. Am. J. Phys. Med. Rehabil..

[49] Floreani M., Rejc E., Taboga P., Ganzini A., Pišot R., Šimunič B., Biolo G., Reggiani C., Passaro A., Narici M. (2018). Effects of 14 days of bed rest and following physical training on metabolic cost, mechanical work, and efficiency during walking in older and young healthy males. PLoS ONE.

[50] Ammarullah M.I., Santoso G., Sugiharto S., Supriyono T., Kurdi O., Tauviqirrahman M., Winarni T.I., Jamari J. (2022). Tresca stress study of CoCrMo-on-CoCrMo bearings based on body mass index using 2D computational model. J. Tribol..

